# Explainable Machine Learning for COVID-19 Pneumonia Classification With Texture-Based Features Extraction in Chest Radiography

**DOI:** 10.3389/fdgth.2021.662343

**Published:** 2022-01-17

**Authors:** Luís Vinícius de Moura, Christian Mattjie, Caroline Machado Dartora, Rodrigo C. Barros, Ana Maria Marques da Silva

**Affiliations:** ^1^Medical Image Computing Laboratory, School of Technology, Pontifical Catholic University of Rio Grande do Sul, PUCRS, Porto Alegre, Brazil; ^2^Graduate Program in Biomedical Gerontology, School of Medicine, Pontifical Catholic University of Rio Grande do Sul, PUCRS, Porto Alegre, Brazil; ^3^Machine Learning Theory and Applications Lab, School of Technology, Pontifical Catholic University of Rio Grande do Sul, PUCRS, Porto Alegre, Brazil

**Keywords:** coronavirus, radiomics, radiological findings, X-rays, machine learning, explainable models, SHAP

## Abstract

Both reverse transcription-PCR (RT-PCR) and chest X-rays are used for the diagnosis of the coronavirus disease-2019 (COVID-19). However, COVID-19 pneumonia does not have a defined set of radiological findings. Our work aims to investigate radiomic features and classification models to differentiate chest X-ray images of COVID-19-based pneumonia and other types of lung patterns. The goal is to provide grounds for understanding the distinctive COVID-19 radiographic texture features using supervised ensemble machine learning methods based on trees through the interpretable Shapley Additive Explanations (SHAP) approach. We use 2,611 COVID-19 chest X-ray images and 2,611 non-COVID-19 chest X-rays. After segmenting the lung in three zones and laterally, a histogram normalization is applied, and radiomic features are extracted. SHAP recursive feature elimination with cross-validation is used to select features. Hyperparameter optimization of XGBoost and Random Forest ensemble tree models is applied using random search. The best classification model was XGBoost, with an accuracy of 0.82 and a sensitivity of 0.82. The explainable model showed the importance of the middle left and superior right lung zones in classifying COVID-19 pneumonia from other lung patterns.

## Introduction

The coronavirus disease-2019 (COVID-19) is a viral respiratory disease with high rates of human-to-human contagious and transmission and was first reported in 27 patients with pneumonia of unknown etiology on December 31st, 2019, in Wuhan, China. The causative agent, a beta coronavirus 2b lineage (1) named severe acute respiratory syndrome coronavirus 2 (SARS-CoV-2), was identified on January 7, 2020, by throat swab samples (2, 3). In December 2020, 1 year after the outbreak, the WHO reported 66,243,918 confirmed cases and 1,528,984 deaths by COVID-19 (4). On January 30, 2021, 1 year after WHO declared COVID-19 as an international public health emergency, confirmed cases achieved 101,406,059 and 2,191,898 deaths (4). August 2021, the mark of 198,778,175 confirmed cases was achieved, with 4,235,559 deaths related to COVID-19 worldwide (4). In August 2020, at least five SARS-CoV-2 virus clade mutations were reported, which have increased the infectivity and viral loads in the population (5). One year later (August 2021), with more than 3,886,112,928 COVID-19 vaccines applied, the third wave of COVID-19 is being noticed in Europe due to the quick virus adaptations increasing the transmissibility and viral load, with four variants of concern and more than ten others identified (6).

The clinical aspects of COVID-19 are highly variable between individuals, varying in different levels of involvement from asymptomatic to lethal conditions. The incubation period goes from 2 to 14 days. A mildly symptomatic condition usually presents fever, dry cough, fatigue, muscle pain, and taste and smell changes, with few patients showing neurological and digestive symptoms (7). Severe symptomatic patients can develop dyspnea, acute respiratory distress syndrome, septic shock, and metabolic acidosis (1). In the United States of America (USA), the younger population, between 18 and 29 years old, are part of the group age with more cases of COVID-19, followed by people between 50 and 64 years (8). However, the number of deaths is higher in the elderly population. Over 30% of deaths are concentrated in 85+ years population, ~60% between 50 and 84 years, and only 0.5% in youngers (18–29 years) (8). COVID-19 incidence is also higher in women (52% of cases), while men show to be more at risk of death (~54%) (8). A study published in August 2020 (1), with 121 Chinese patients with COVID-19, showed that the risk of adverse outcomes in individuals with more than 65 years is 2.28 times higher, and initial clinical manifestation does not differ between non-severe and severe cases, as in survivors and non-survivors. The risk factors for predicting COVID-19 severity were cardiovascular and cerebrovascular diseases (1). Higher lactate dehydrogenase (LDH) and coagulation dysfunction also contribute to the severity of the disease and progression to death (1).

The diagnosis of COVID-19 uses two approaches: reverse transcription-PCR (RT-PCR) (7) and chest X-rays (CXRs) (9). However, with the possibility of RT-PCR false-negative results, clinical and laboratory tests have usually been added to the patient diagnosis investigation and imaging findings. The imaging techniques are typically CXR or CT, and the findings have been compared with those of typical pneumonia. COVID-19 pneumonia does not have a defined set of imaging findings resulting in heterogeneous positivity definitions. The diagnosis sensitivity ranges for chest CT and CXR are from 57.4 to 100% and 59.9 to 89.0%, specificity from 0 to 96.0 and 11.1 to 88.9%, respectively (10). Automatic methods of prediction of COVID-19 in CT have been evaluated in several articles, as described by Chatzitofis et al. (11) and Ning et al. (12). Even CT provides higher image resolution, CXR images are less costly, available in clinics and hospitals, implies a lower radiation dose, and have a smaller risk of contamination of the imaging equipment and interruption of radiologic services to decontamination (13, 14). Also, abnormality findings on CT are mirrored in CXR images (14, 15).

The interpretation of radiological lung patterns can reveal differences in lung diseases. For COVID-19-related pneumonia, the characteristic pattern in CXR includes a pleuropulmonary abnormality with the presence of bilateral irregular, confluent, or bandlike ground-glass opacity or consolidation in a peripheral and mid-to-lower lung zones distribution with less likely pleural effusion (14, 16). For other lung diseases, like typical pneumonia, radiological patterns are related to the disease origin: bacterial, viral, or another etiology. In general, CXR findings show segmental or lobar consolidation and interstitial lung disease (17). Specifically, for viral pneumonia caused by adenovirus, the radiological pattern is characterized by multifocal consolidation or ground-glass opacity. In addition, there are bilateral reticulonodular areas of opacity, irregular or nodular regions of consolidation for pneumonia by influenza virus (18). For bacterial pneumonia, there are three main classifications due to the affected region: lobar pneumonia, with confluent areas of focal airspace disease usually in just one lobe, bronchopneumonia, with a multifocal distribution with nodules and consolidation in both lobes, and acute interstitial pneumonia that involves the bronchial and bronchiolar wall, and the pulmonary interstitium (19).

Apart from typical visual interpretation, the lung disease patterns can be studied through texture-feature analysis and radiomic techniques. However, due to radiologists' unfamiliarity with COVID-19 patterns, computer-aided diagnosis (CAD) systems help to differentiate COVID-19-related and other lung patterns.

Radiomics is a natural extension of CAD that converts the medical images into mineable high-dimensional data, allowing hypothesis generation, testing, or both (20). Computer-based texture analysis can be present in radiomics and reflects the tissue changes quantitatively from a healthy state to a pathological one. The extracted features can feed a classification model. The process has been widely explored to help radiologists achieve a better and faster diagnosis and is being applied to analyze and classify medical images to detect several diseases such as skin cancer (21), neurological disorders (22), and pulmonary diseases, like cancer (23) or pneumonia (24). For example, COVID-19-related pneumonia studies have been using various methods to differentiate the disease from typical pneumonia healthy individuals or even several lung diseases. Many approaches use deep learning (DL) framework, basing the feature extraction and classification in convolutional neural networks (CNN) models (25–28). However, DL models are not inherently interpretable and cannot explain their predictions intuitively and understandably. Hand-crafted feature extraction yet has mathematical definitions and can be associated with known radiological patterns.

The interpretability of results using medical images has been highly required. Therefore, explainable AI (Artificial Intelligence) predictions have been developed, such as the Shapley Additive Explanations (SHAP) framework (29). Our goal is to investigate the radiomic features and classification models to differentiate chest X-ray images of COVID-19-based pneumonia and diverse types of lung pathologies. We aim to provide grounds for understanding the distinctive radiographic features of COVID-19 using supervised ensemble machine learning methods based on trees in an interpretable way using the SHAP approach to explain the meaning of the most important features in prediction. Our study analyzes the lung in three zones in both lobes, showing the middle left and superior right zones' importance in identifying COVID-19.

## Materials and Methods

We retrospectively used a public dataset of CXR images of COVID-19-related pneumonia and lung images of patients with no COVID-19 to investigate the radiomic features that can discriminate COVID-19 from other lung radiographic findings. We extracted first- and second-order radiomic features and divided our analysis into two main steps. First, we performed k-folds cross-validation to select the algorithm with the best performance classifying COVID-19 pneumonia and non-COVID-19. Second, we performed an explanatory approach to select the best set of radiomic features that characterize COVID-19 pneumonia.

### Image Dataset

**Figure 1:**
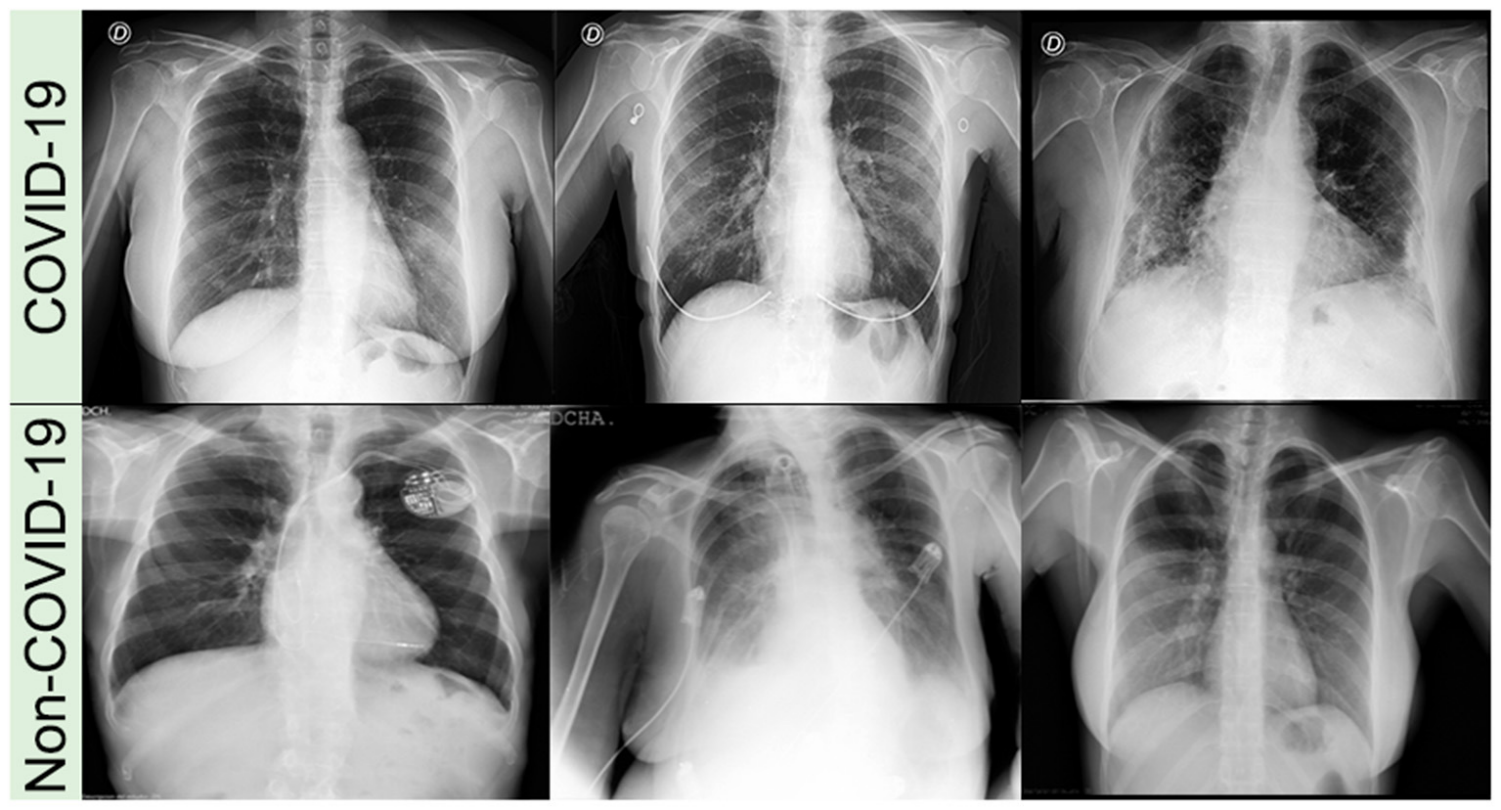
Examples of CXR images from COVID-19 and non-COVID-19 datasets.

We used two public multi-institutional databases related to COVID-19 and non-COVID-19 to train and evaluate our model. BIMCV (Valencian Region Medical ImageBank) is a large dataset, with annotated anonymized X-ray and CT images along with their radiographic findings, PCR, immunoglobulin G (IgG), and immunoglobulin M (IgM), with radiographic reports from Medical Imaging Databank in Valencian Region Medical Image Bank. BIMCV-COVID+ (30) comprises 7,377 computed radiography (CR), 9,463 digital radiography (DX), and 6,687 CT studies, acquired from consecutive studies with at least one positive PCR or positive immunological test for SARS-Cov-2. BIMCV-COVID- (31) has 2,947 CR, 2,880 DX, and 3,769 CT studies of patients with negative PCR and negative immunological tests for SARS-Cov-2. Both databases have all X-ray images stored in 16-bit PNG images in their original high-resolution scale, with sizes varying between 1,745 × 1,465 pixels and 4,248 × 3,480 pixels for patients with COVID-19 and between 1,387 × 1,140 pixels and 4,891 × 4,020 pixels for non-COVID-19. Figure 1 shows an example of CXR images of both datasets (BIMCV-COVID+ and BIMCV-COVID–).

We used only CXR images of anteroposterior (AP) and posteroanterior (PA) projections of adult patients (≥18 years old). Some images from the dataset do not have information regarding projection (AP, PA, or lateral) in the DICOM tag, and some projection tags are mislabeled. All images with missing the projection DICOM tag were discarded and, from the selected AP or PA projections, they were visually inspected and discarded if mislabeled.

For COVID-19 positive patients, we selected only the first two images between the first and last positive PCRs. For non-COVID-19 participants with more than two X-ray acquisitions, only the first two images were selected. Therefore, we have 2,611 images from patients with COVID-19, and we randomly chose 2,611 images from non-COVID-19 to ensure a balanced dataset. Demographic information regarding selected patients is shown in Table 1. In addition, since some CXR images were stored with an inverted lookup table, images with photometric interpretation equal to “monochrome1” were multiplied by minus one and summed with their maximum value to harmonize the dataset.

**Table 1:** Demographic data of our study.

	**COVID-19**	**Non-COVID-19**
Age	62 ± 16	64 ± 19
Sex (male)	1,358	1,268
Sex (female)	1,253	1,343

Previous studies already used the BIMCV-COVID dataset to evaluate lung segmentation (32), data imbalance corrections (33), DL classification models (34–36), and other imaging challenges (37, 38).

### Lung Segmentation

We applied a histogram equalization in each CXR image (39, 40) to normalize the intensity values and reduce the dataset's features variability. Pixel values were normalized to 8-bits per pixel and resampled to 256 × 256 pixels. We segment lungs using an open-source pretrained U-Net-inspired architecture segmentation model to generate lung masks^1^. The model was trained in two different open CXR databases: JSRT (Japanese Society of Radiological Technology) (41) and Montgomery County (42). The databases used for training came from patients with tuberculosis, so they are not specific for COVID-19-based pneumonia lung segmentation.

We applied the opening morphological operator in each mask to remove background clusters and fill holes of the resulting lung mask, using a square structuring element, 8-connected neighborhood. The opening morphological operation smooths an object's contour, breaks narrow isthmuses, and eliminates thin protrusions. The mathematical details of opening morphological operation can be found in Gonzalez and Woods (43).

We removed all clusters with <5 pixels and all connected regions with <75 pixels. The lung mask is stretched back to the original image size and applied to the original image before processing. We normalized all segmented lung images considering only pixels inside the lung mask, between 0 and 255.

**Figure 2:**
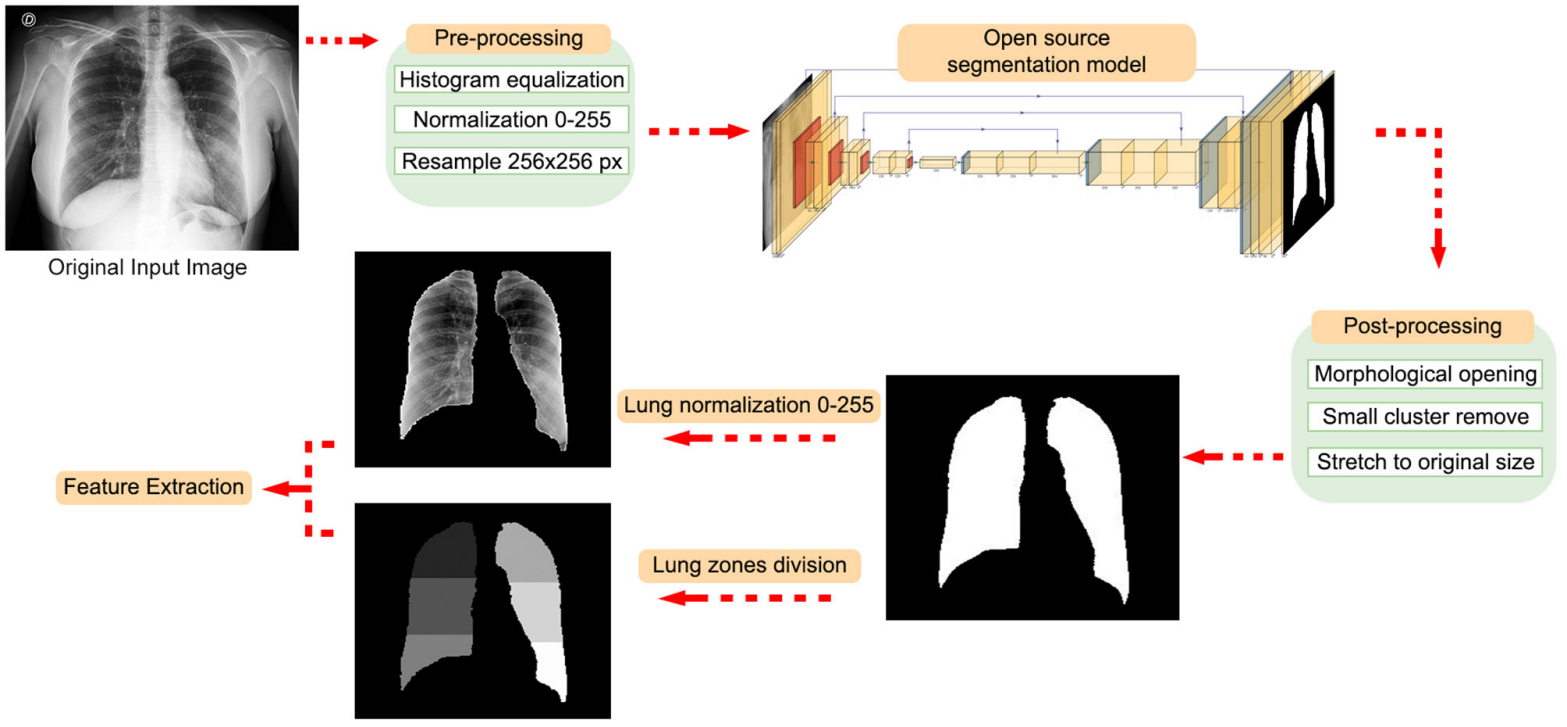
Workflow of the segmentation process.

We split the image between the left and right side using the centroid of two areas; if the centroid is located within the first half of the matrix size (from left to right), it is considered as part of the right lung (the radiological image in CXR is mirrored). Next, each lung's height is divided into upper, middle, and bottom zones, determined by the extremities' distance, divided into thirds. The segmentation workflow is shown in Figure 2.

### Radiomic Features

We used the PyRadiomics library to extract the first and second-order statistical texture-based features for each lung mask. The mathematical formulation of the features can be found in Zwanenburg, Leger, and Vallières (44). The radiomic features are divided into five classes (45):

*First-order features (18 features)*: These are based on the first-order histogram and related to the pixel intensity distribution.

*Gray-level co-occurrence matrix or GLCM (24 features)*: This gives information about the gray-level spatial distribution, considering the relationship between pixel pairs and the frequency of each intensity within an 8-connected neighborhood.

*Gray-level run length matrix or GLRLM (16 features)*: This is like GLCM; it is defined as the number of contiguous pixels with the same gray level considering a 4-connected neighborhood, indicating the pixel value homogeneity.

*Gray-level size zone matrix or GLSZM (16 features)*: This is used for texture characterization; it provides statistical representation by estimating a bivariate conditional probability density function of the image distribution values and is rotation-invariant.

*Gray-level dependence matrix or GLDM (14 features)*: This quantifies the dependence of gray image level by calculating the connectivity at a certain distance when its difference in pixel intensity is <1.

### Model Selection

We performed 10-folds cross-validation after radiomic feature extraction to guarantee unbiased metrics results and error generalization. We realized that a normalization between 0 and 1 and used SHAP- Recursive Feature Elimination with Cross-Validation (RFECV) feature selection in 9-folds of the dataset. Each machine learning model was trained using the selected features with the hyperparameter optimization method, and randomized search with cross-validation from the sci-kit learn library (46, 47). The method uses a range of values for each parameter in the model. It tests a given number of times with different combinations and splits of training data, measuring the model performance in the validation set. We chose to run 1,000 iterations for each model, using an intern 5-folds stratified cross-validation. The parameter values explored in each model are shown in Table 2. We chose the best parameters based on the best performance of recall in cross-validation. After hyperparameter optimization, model evaluation is performed in the last fold.

**Table 2:** Parameter values explored by random search in XGBoost and Random Forest Classifier models.

**XGBoost**		**Random Forest Classifier**	
min_child_weight	1, 2, 3, 4, 5, 6, 7, 8, 9, 10	n_estimators	10, 50, 100, 200
max_depth	1, 2, 3, 4, 5, 6, 7, 8, 9, 10	max_depth	None, 1, 2, 3, 4, 5, 6, 7, 8, 9, 10
lambda^*^	0–1	criterion	gini, entropy
gamma^*^	0–1	min_sample_split	1, 2, 3, 4, 5, 6, 7, 8, 9, 10
eta^*^	0–1	min_sample_leaf	1, 2, 3, 4, 5
objective	binary:logistic		
tree_method	gpu_hist		

* *10 random values in the range*.

#### Feature Selection

Feature selection is the process of selecting the most relevant features for a given task. The process reduces the computational cost regarding the training and evaluation of the machine learning model and improves the generalization (48). Moreover, some features may be irrelevant or redundant, negatively impacting the modeling, adding biases (49). To avoid these issues, we decided to make a feature selection before our modeling.

We used the SHAP-RFECV from the Probatus python library to perform the feature selection. Further information about the SHAP-RFECV algorithm and its applications can be found on the Probatus webpage^2^. The method uses a backward feature elimination based on the SHAP value of feature importance. The designed model is trained with all features initially and uses cross-validation (10-fold) to estimate each feature's SHAP importance value. At the end of each round, the features with the lowest importance are excluded. Then, the training is done again until the number of features chosen by the user is reached. We decided to remove 20% of the lowest importance features in each iteration for faster reduction of features in early iterations and more precise results in the later ones until it reaches the minimum value of 20 features.

#### Machine Learning Models

We trained two ensemble classification models based on tree-based models using the scikit-learn (47) library and XGBoost (XGB) (50) on Python version 3.6.5. The classification methods used in our article are the XGB and the Random Forest (RF).

XGBoost is a scalable ensemble model based on an extreme gradient for tree boosting. It is based on regression trees, which in contrast to decision trees, contains a continuous score on each of the leaves. The input data is sorted into blocks of columns that are categorized by the corresponding feature value. The split search algorithm runs in the block seeking the split candidates' statistics in all leaf branches. It uses decision rules into trees to determine for each leaf the example will be placed. The final prediction is calculated by summing up the score in the corresponding leaves (51). The proper algorithm and mathematical formulation are addressed in Chen (50).

Random Forest is a tree-based ensemble learning algorithm that induces a pre-specified number of decision trees to solve a classification problem. Each tree is built using a subsample of the training data, and each node searches for the best feature in a subset of the original features. The assumption is that by combining the results of several weak classifiers (each tree) *via* majority voting, one can achieve a robust classifier with enhanced generalization ability. The mathematical formulation of RF is described in Breiman (52).

#### Performance Evaluation

Accuracy, sensitivity, precision, F1-Score, and the area under the curve (AUC) of the receiver operating characteristic (ROC) were used for model evaluation. The final model was selected based on the best sensitivity achieved in the cross-validation. Each metric is calculated as follows (53):


$$\begin{array}{l} \;Accuracy=\frac{TP+TN}{TP+TN+FP+FN}\\ \;Sensitivity=\frac{TP}{TP+FN}\\ \;Precision=\frac{TP}{TP+FP}\\ F1\;Score=2\times \frac{precision\times sensitivity}{precision+sensitivity} \end{array}$$

where: TP = true positive, TN = true negative, FP = false positive, and FN = false negative.

### Explanatory Approach

Despite their complexity, approaches to making AI models “interpretable” have gained attention to enhance the understanding of machine learning algorithms. Explaining tree models is particularly significant because the pattern that the model uncovers can be more important than the model's prediction itself. The SHAP approach is an additive feature attribution method that assigns an “importance value” to each feature for a particular prediction (29, 54). The SHAP approach satisfies three important properties for model explanation: (i) local accuracy because the explanation model and the original one has to match, at least, the output for a specific input; (ii) consistency, because if the model changes, it is because a feature's contribution increases or stay the same regardless of other inputs, so the input's attribution should not decrease; and (iii) missingness, meaning the missing values of features in the original input have no impact.

In our work, we chose the SHAP approach with tree models because it is calculated in each tree leaf and gives interpretability for local explanations, which reveals the most informative features for each subset of samples. Local explanations allow the identification of global patterns on data and verify how the model depends on its input features. It also increases the signal-to-noise ratio to detect problematic data distribution shifts, making it possible to analyze the behavior of the entire dataset, which is composed of medical images from different databases (54).

The SHAP approach uses an extension of Shapley values from the game theory to calculate the feature importance. The SHAP values are calculated based on the prediction difference when using all features and when using just a few ones. It addresses how the addition of one feature improves or not the prediction (55). In tree-based models, the SHAP values are also weighted by the node sizes, meaning the number of training samples in the node. Finally, the feature importance assumes that the features with large absolute SHAP values have more importance than others with smaller absolute values. To access the global importance, we average the feature importance across all data (56).

We use the SHAP-RFECV approach on the final model to evaluate the most important features and how they affect the model's prediction.

## Results

We extracted 88 features for each lung zone. Next, we applied RF and XGB models to analyze the performance with 10-fold cross-validation. Table 2 shows the parameters used for both models, and Table 3 has the performance metrics.

**Table 3:** Mean cross-validation metrics of XGBoost and Random Forest models.

**Model**	**Accuracy**	**F1-Score**	**Sensitivity**	**Precision**
XGB	0.82	0.82	0.82	0.82
RF	0.77	0.78	0.81	0.75

The XGBoost model was selected for hyperparameters optimization and feature selection using SHAP due to its higher classification performance. Table 4 shows the hyperparameters, and Table 5 shows the selected features.

**Table 4:** Hyperparameters for XGBoost.

**XGBoost**	
min_child_weight	6
max_depth	3
lambda	1
gamma	0.50230907476997
eta	0.50515969241175
objective	binary:logistic
tree_method	hist

**Table 5:** Chosen features by XGBoost model.

**XGBoost**	**Abbreviation**
Bottom Left - First Order - Maximum	BL-1st-M
Bottom Right - First Order - Energy	BR-1st-E
Bottom Right - First Order - Kurtosis	BR-1st-K
Bottom Right - GLCM - Cluster Prominence	BR-GLCM-CP
Bottom Right - GLCM - Difference Variance	BR-GLCM-DV
Middle Left - First Order - Kurtosis	ML-1st-K
Upper Left - First Order - Range	UL-1st-R
Upper Left - GLCM - Idmn	UL-GLCM-LDMN
Upper Left - GLRLM - Run Entropy	UL-GLRLM-RE
Upper Right - First Order - Robust Mean Absolute Deviation	UR-1st-RMAD
Upper Right - GLCM - Cluster Prominence	UR-GLCM-CP
Upper Right - GLCM - Cluster Shade	UR-GLCM-CS
Upper Right - GLCM - MCC	UR-GLCM-MCC
Upper Right - GLRLM - Gray Level Non-Uniformity	UR-GLRLM-GLNU
Upper Right - GLRLM - High Gray Level Run Emphasis	UR-GLRLM-HGLRE
Upper Right - GLSZM - Gray Level Non-Uniformity Normalized	UR-GLSZM-GLNUN
Upper Right - GLSZM - Gray Level Variance	UR-GLSZM-GLV
Upper Right - GLSZM - Large Area High Gray Level Emphasis	UR-GLSZM-LAHGLE
Upper Right - GLSZM - Size Zone Non-Uniformity	UR-GLSZM-SZNU
Upper Right - GLSZM - Small Area High Gray Level Emphasis	UR-GLSZM-SAHGLE

**Figure 3:**
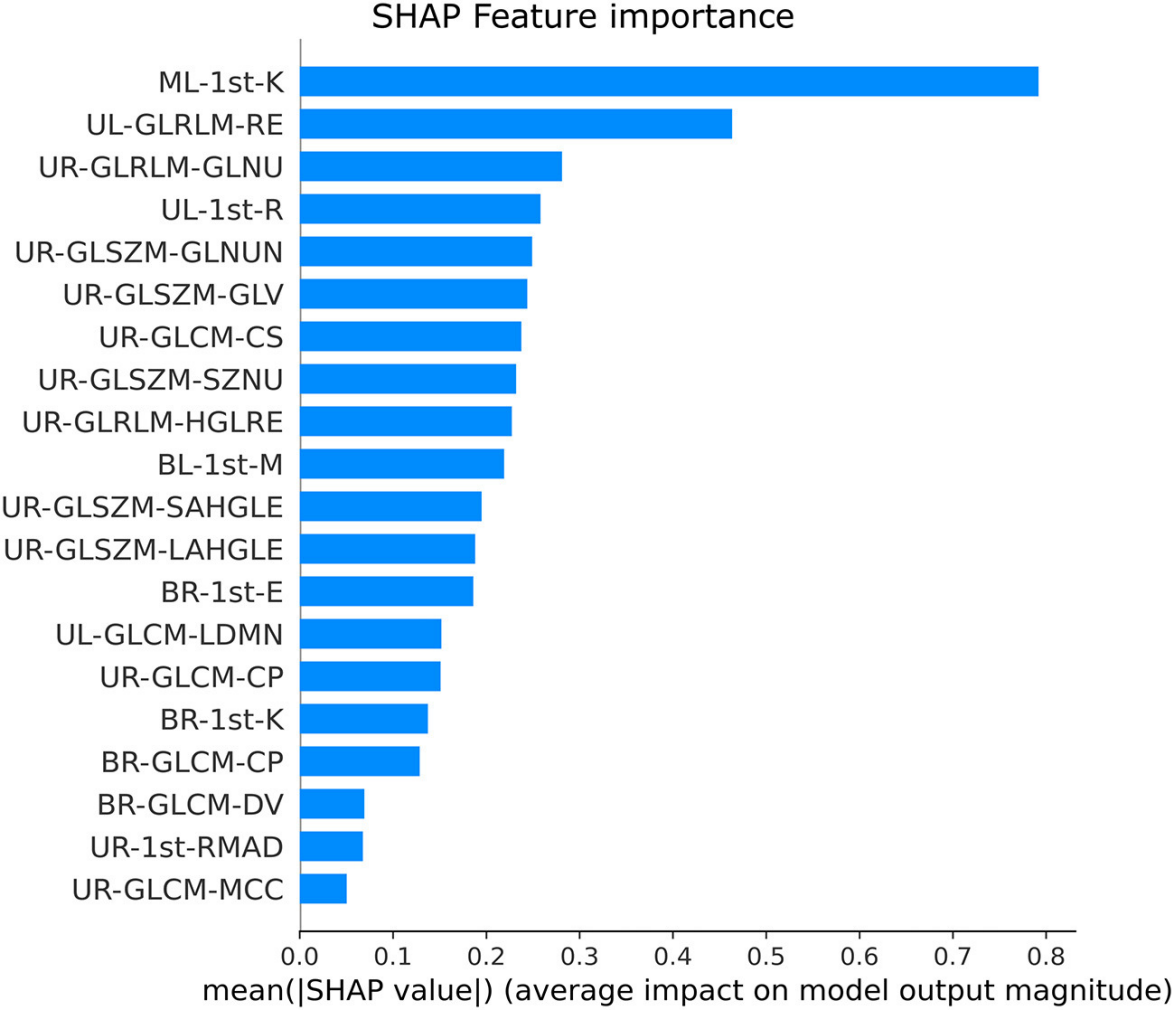
Feature importance for each radiomic feature with the XGBoost model.

**Figure 4:**
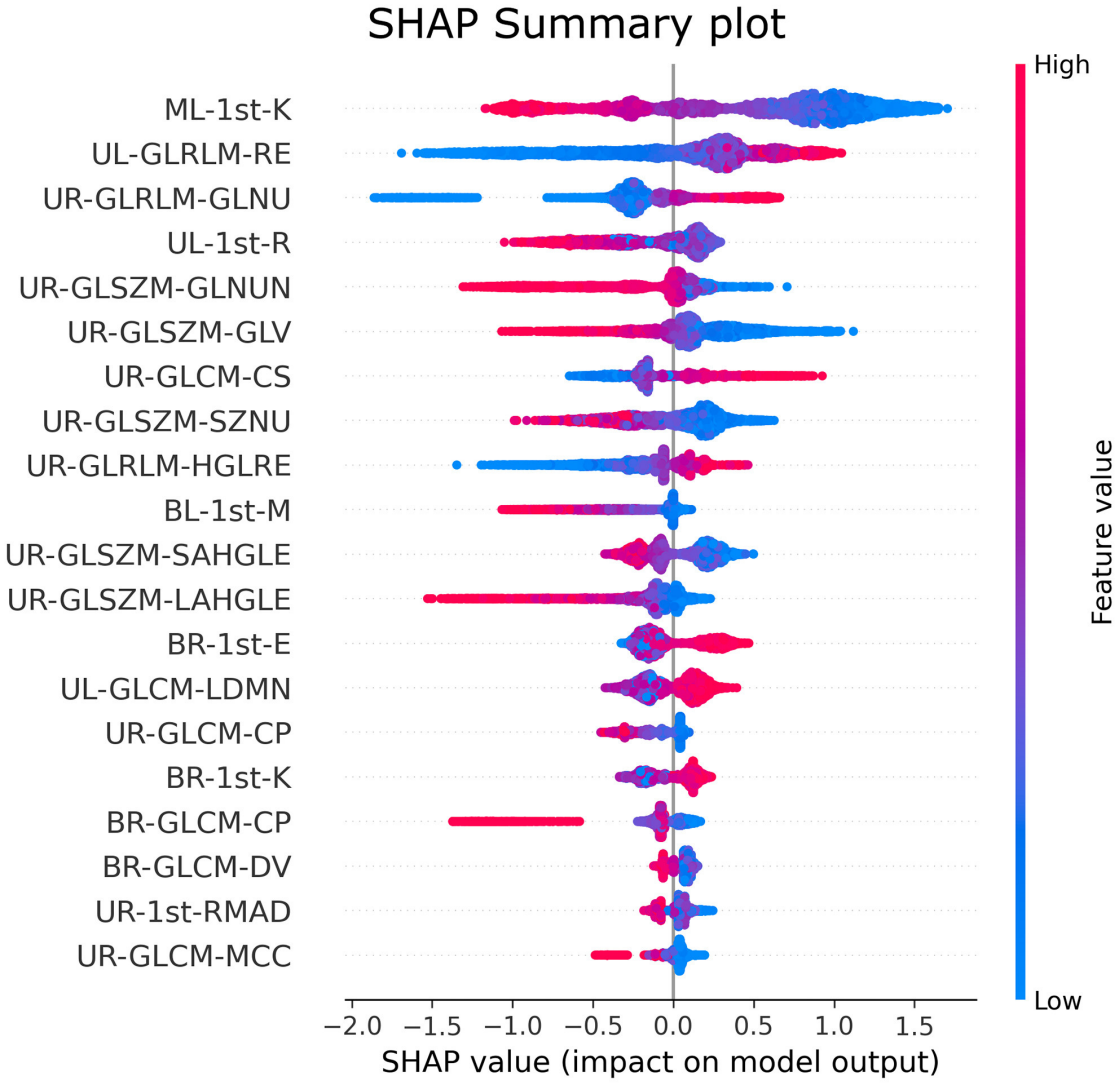
Impact of important features in the XGBoost model output.

The SHAP feature importance values are shown in Figure 3 for the 20 selected features. Figure 4 shows the effect of each feature in the model prediction.

**Figure 5:**
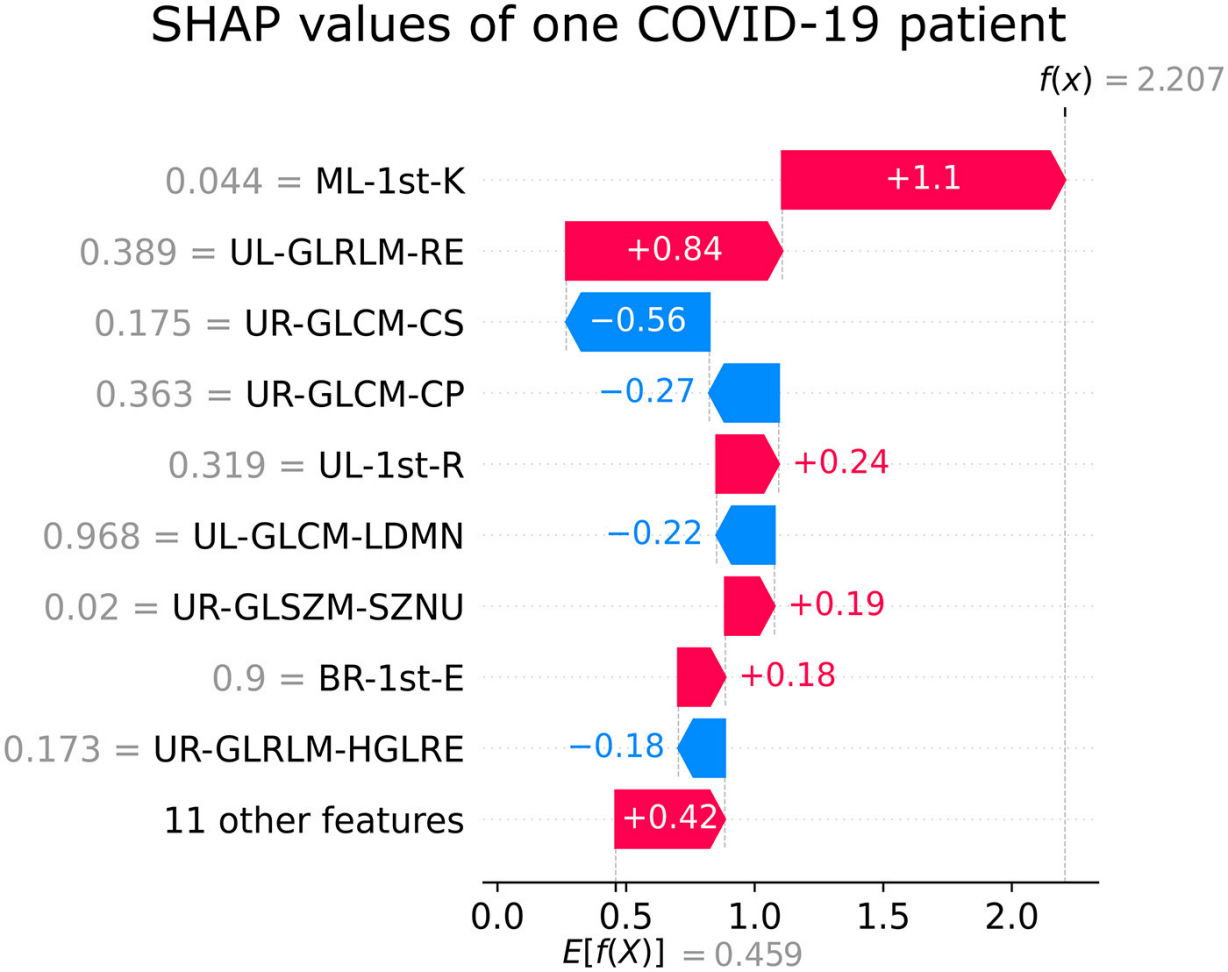
Example of SHAP values affecting XGBoost model output for a single COVID-19 CXR image.

**Figure 6:**
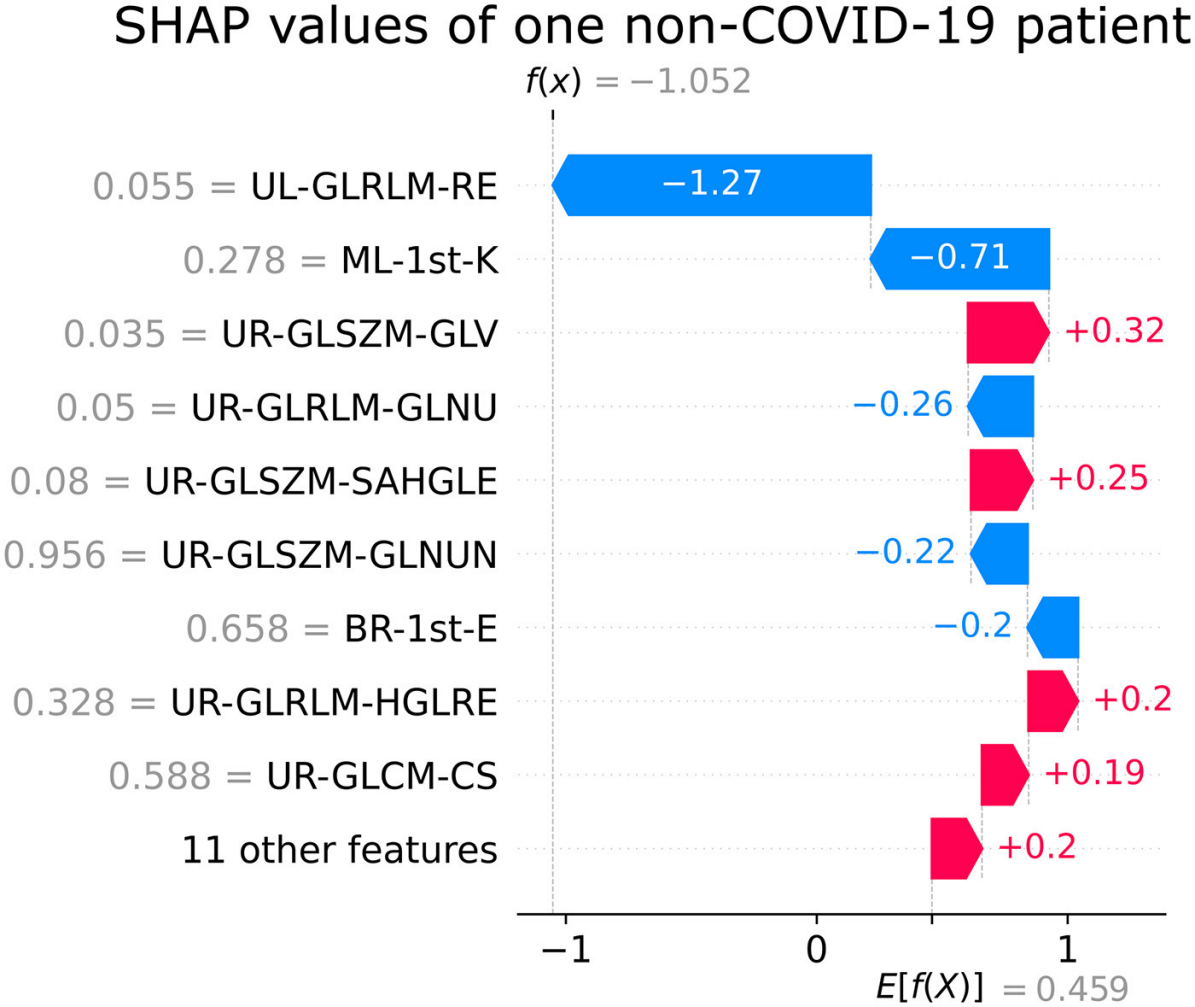
Example of SHAP values affecting XGBoost model output for a single non-COVID-19 CXR image.

Figures 5, 6 show the decision's plot of one COVID-19 pneumonia case and one non-COVID-19 pneumonia case, respectively. The plot shows the SHAP values related to each feature's importance and how they predict the classification for two random individuals. For example, the positive SHAP value in Figure 5, *f*_(x)_ = 2.207, is related to the model prediction identifying the CXR as a COVID-19. Similarly, the negative SHAP value in Figure 6, *f*_(x)_ = −1.052, is related to the model prediction identifying the CRX as a patient with non-COVID-19.

## Discussions

In the latest year, numerous studies have been developed applying different computer-aided methods to aid in diagnosing and in the prognosis COVID-19 (11, 12, 14, 15, 25–28, 57–75). However, most studies do not use explainable methods (57). Our approach uses the hand-crafted radiomics features approach and ensemble tree-based machine learning classification models to differentiate COVID-19-induced pneumonia from other lung pathologies and healthy lungs in CXR images. We use ensemble tree-based models since they are more accurate than artificial neural networks in many applications (54). Looking for explainability, we use the SHAP approach to unveil why specific features with low or high SHAP values are associated with the disease. Moreover, we choose to analyze different lung zones, previously segmented, looking for regions of interest in the disease.

The computer-aided methods using non-segmented images may lead to biases (58, 59) since the models may associate elements from outside the lungs, such as bones and muscles, not related to the disease, with the presence of COVID-19. Restricting the region of interest ensures that the features extracted are associated with the radiological information present in the lung zone. Our study uses automatic lung segmentation and divides the lung into six zones, which are independently analyzed. The approach allows small structures in the analysis, which could be suppressed by analyzing the entire lung at once.

Nowadays, COVID-l9 radiological studies are focused on CT findings, which have better sensitivity than CXR. However, CT is more expensive and scarcer than conventional X-rays, requiring complicated decontamination after scanning patients with COVID-19. Therefore, the American College of Radiology (60) recommends CT to be used sparingly and reserved for hospitalized patients with COVID-l9 symptomatic with specific clinical indications. A portable chest X-ray equipment is suggested as a viable option to minimize the risk of cross-infection and avoid overload and disruption of radiological departments. Moreover, studies have shown that CXR COVID-19 findings mirror the CT findings (14, 15), with less radiation dose and higher availability in clinics and hospitals.

The use of feature importance techniques can improve clinical practice in different medical fields. Hussain et al. (76) showed the benefits of multimodal features extracted from congestive heart failure and normal sinus rhythm signals. The application of feature importance ranking techniques was beneficial to distinguish healthy subjects from those with heart failure. The same group also found that the use of the synthetic minority oversampling technique can improve the model performance when dealing with imbalanced datasets (77).

### Meaningful Texture-Based Features in COVID-19 Pneumonia

The main contribution of this study is the findings of a group of meaningful radiomic features in differentiating COVID-19 from other lung diseases using CXR using an explainable machine learning approach. The most relevant features are presented in Figure 3. In addition, SHAP values summary plots can be used to try and explain how each feature is increasing or decreasing the model output, meaning the probability of classifying a CXR image as COVID-19 pneumonia or not.

As seen in Figure 3, the first-order kurtosis on the middle-left lung was, by far, the feature with the highest importance for classification. Kurtosis is, in general, a measure of the “peakedness” of the distribution of the values (78). Since it is a first-order feature, it is directly related to the pixel values on the CXR image. COVID-19 induces consolidation and ground-glass opacification, which increases these pixel values in the lung region and may induce a distribution with lighter tails and a flatter peak, resulting in lower kurtosis values (78). These lower values were associated with the disease, as can be seen in Figure 4.

In CXR images, the heart partially overlaps with the lungs in the left middle region, influencing feature importance. Despite being the most important feature, kurtosis was the only feature selected from this region. The second and third most important features are both related to gray-level homogeneity. Higher values of Run Entropy in GLRLM, which were associated with COVID-19 in the upper left region, indicate more heterogeneity in the texture patterns. Higher values of gray-level non-uniformity in GLRLM in the upper right region, also associated with the disease, indicate lesser similarity between intensity values, corroborating the previous feature findings. COVID-19-induced consolidations tend to be diffuse or patchy, which may explain these features associations (70).

We won't go over other features individually due to their decreasing and similar importance. However, it is important to note that from the 20 selected features, half are extracted from the upper right zone. Our previous study (61) showed similar results, where the two most important features were also from the upper right lung region. Moreover, no middle right zone features were selected.

### Comparison With Related Work

Saha et al. (62) created EMCNet, an automated method to diagnose COVID-19 and healthy cases from CXR images. Their method uses a simple CNN to extract 64 features from each image and then classify binary with an ensemble of classifiers composed of Decision Tree, Random Forest, Support Vector Machines, and AdaBoost models. They used 4,600 images (2,300 COVID-19 and 2,300 healthy) from different public datasets (38, 79–81) and applied resizing and data normalization. As a result, they achieved 0.989 for accuracy, 1.00 for precision, 0.9782 for sensibility, and 0.989 in F1-score. In comparison with our study, we both use almost the same number of CXR images, but we only use two databases, mostly from the same facilities. However, the authors' data included pediatrics patients, leading to biases due to age-related characteristics. Duran-Lopez et al. (34) proposed COVID-XNet, a DL-based system that uses pre-processing algorithms to feed a custom CNN to extract relevant features and classify between COVID-19 and normal cases. Its system achieved 92.53% sensitivity, 96.33% specificity, 93.76% precision, 93.14% F1-score, 94.43% balanced accuracy, and an AUC value of 0.988. Class Activation Maps were used to highlight the main findings in COVID-19 X-ray images and were compared and verified with their corresponding ground truth by radiologists.

Cavallo et al. (63) made a texture analysis to evaluate COVID-19 in CXR images. They used a public database selecting 110 COVID-19-related and 110 non-COVID-19-related interstitial pneumonia, avoiding the presence of wires, electrodes, catheters, and other devices. Two radiologists manually segmented all the images. After normalization, 308 textures were extracted. An ensemble made by Partial Least Square Discriminant Analysis, Naive Bayes, Generalized Linear Model, DL, Gradient Boosted Trees, and Artificial Neural Networks models achieved the best results. The ensemble model performance was 0.93 of sensitivity, 0.90 of specificity, and 0.92 of accuracy. We have not attained these high metrics. However, we used much more images (5,222 vs. 220), and like in the previous study, they used mixed-age data, where COVID-19 images were retrieved from adult patients and non-COVID-19 images from pediatric ones.

Rasheed et al. (64) proposed a machine learning-based framework to diagnose COVID-19 using CXR. They used two publicly available databases with a total of 198 COVID-19 and 210 healthy individuals, using Generative Adversarial Network data augmentation to get 250 samples of each group. Features were extracted from 2D CXR with principal component analysis (PCA). Training and optimization were done with CNN and logistic regression. The PCA with CNN gave an overall accuracy of 1.0. Using just 500 CXR images from COVID-19 and non-COVID-19 individuals from two different datasets and training the model with data augmentation techniques may suggest the possibility of overfitting or that classification can be differentiating the two datasets and not the disease itself.

Brunese et al. (65) developed a three-phase DL approach to aid in COVID-19 detection in CXR: detect the presence of pneumonia, discern between COVID-19 induced and typical pneumonia, and localize CXR areas related to COVID-19 presence. Different datasets for different pathologies were used, two datasets of patients with COVID-19 and one of the other pathologies. In total, 6253 CXR images were used, but only 250 were from patients with COVID-19. Accuracy for pneumonia detection and COVID-19 discrimination was 0.96 and 0.98, respectively. Activation maps were used to verify which parts of the image were used by the model for classification. They showed a high probability of prediction in the middle left and upper right lungs, agreeing with our findings.

Kikkisetti et al. (66) used portable CXR from public databases with CNN and transfer learning to classify the images between healthy, COVID-19, non-COVID-19 viral pneumonia, and bacterial pneumonia. They used two approaches, using all CXR and only segmented lungs. CNN heatmaps showed that with the whole CXR, the model used outside the lungs information to classify. It is a tangible example of the importance of segmenting the CXR images, especially when using data from different locations, to avoid biases created by the annotations in X-rays. They achieved an overall sensitivity, specificity, accuracy, and AUC of 0.91, 0.93, 0.88, and 0.89, respectively, with segmented lungs. However, they used pediatric data mixed with adults. It is interesting to note that in their CNN heatmaps, the lower and middle portion of the left lung showed a high importance in their classification, in agreement with our results. We have three features from these locations, which are essential in the COVID-19 classification, and two were selected from these regions. However, one feature is, by far, the most important for classification. Moreover, our database is almost five times larger.

Yousefi et al. (67) proposed a computer-aided detection of COVID-19 with CXR imaging using deep and conventional radiomic features. A 2D U-net model was used to segment the lung lobes. They evaluated three different unsupervised feature selection approaches. The models were trained using 704 CXR images and independently validated using a study cohort of 1,597 cases. The resulting accuracy was 72.6% for multiclass and 89.6% for binary-class classification. Since unsupervised models were used, it is impossible to check if the most important features are like ours. Unfortunately, the lobes were not investigated separately for further comparisons.

Casiraghi et al. (82) developed an explainable prediction model to process the data of 300 patients with COVID-19 to predict their risk of severe outcomes. They collected clinical data and laboratory values. The radiological scores were retrospectively evaluated from CXR by either pooling radiologists' scores or applying a deep neural network. Boruta and RF were combined in a 10-fold cross-validation scheme to produce a variable importance estimate. The most important variables were selected to train an associative tree classifier, with AUC 0.81–0.76, sensitivity 0.72–0.66, F1 score 0.62–0.55, and accuracy from 0.74 to 0.68. The PCR achieved the highest relative relevance, together with the patient's age and laboratory variables. They noted that the radiological features extracted from radiologists' scores and deep network were positively correlated, as expected. Moreover, their radiological features were also correlated with PCR and have an inverse correlation with the saturation values. However, they did not evaluate radiomic features extracted from CXR for comparison with our work.

Even though the image characteristics are different in CT and CXR, they share the same physical interaction with tissues using X-rays. Caruso et al. study (68) used CT texture analysis to differentiate patients with positive COVID-19 and negative ones. Sensitivity and specificity were 0.6 and 0.8, respectively. The feature with the highest correlation with patients with positive COVID-19 compared with negative ones was kurtosis, with lower values associated with the disease. In our work, the most important classification feature was first-order kurtosis with the same lower values behavior associated with the disease.

Similarly, Lin et al. (69) developed a CT-based radiomic score to diagnose COVID-19 and achieve a sensibility of 0.89. They also found that the GLCM MCC feature was important during the classification. In our study, we also found the same feature in the upper right lung. Finally, Liu et al. (70) analyzed the classification performance in CT images using two approaches (only clinical features and clinical with texture features), increasing their sensitivity to 0.93. Their results show the importance of clinical information, if available. They also found cluster prominence features as important, but their analysis was made using a wavelet filter.

Shiri et al. (71) made an analysis using CT images and clinical data to develop a prediction model of patients with COVID-19. The model with the highest performance achieves a sensibility and accuracy of 0.88. Interestingly, one of the radiomic features used in their work, GLSZM – SAHGLE, was also selected in our model in the upper right lung zone.

### Limitations

One of the main limitations of AI studies is the currently available COVID-19 and non-COVID-19 CXR databases (72). Even though databases have many data, most have several missing and mislabeled data. Moreover, most COVID-19 public databases do not include non-COVID images from the same medical center, requiring other databases from different facilities. Medical centers use various scanners and protocols, leading to different image patterns if no previous harmonization is executed. In studies using multiple databases with other pathologies, computer-aided methods may learn to differentiate the database pattern rather than the lung pathologies (73). Finally, a limitation of the databases used in this work is they do not include clinical data from patients.

The most important concern about some studies is that some databases of typical pneumonia CXR have images from pediatric and adult patients. They are primarily used due to the differentiation between viral and bacterial pneumonia. However, this may increase biases due to age-related characteristics (71). Usually, imaging acquisition protocols for pediatric patients are made with less radiation due to radiological protection.

Our model reached an accuracy of 0.82, a sensitivity of 0.82, a precision of 0.82, and F1-score metrics of 0.82 using CXR images and SHAP RFECV. Other COVID-19 studies using CXR images and machine learning models reached accuracy between 0.92 and 0.99, and sensibility between 0.91 and 0.99 (62, 63). However, the limitations of the studies with higher scores discussed in the previous section should be considered; some studies used non-segmented images (62–64, 66), and other studies used data from pediatric patients (62, 64, 65) (1–5 years old) mixed with adult data.

The main limitations of our study are the absence of clinical data to improve our models and the lack of statistical analysis to have more confidence about the importance of the features. Moreover, we limited the features extraction and other features that could be included, like the neighboring gray-tone difference matrix. Finally, we did not evaluate the effect of features' extraction applying different pre-processing filters.

### Future Directions

The sudden onset of COVID-19 generated a global task force to differentiate it from other lung diseases. With the main symptoms related to atypical pneumonia, the number of CXR and chest CT datasets has rapidly increased. More than 1 year and a half from the COVID-19 onset, the available datasets of chest images are more extensive, so it is possible to have confidence in classifying the disease from other pulmonary findings. However, before the 2020 pandemic, most lung radiomic signature analysis studies focused on identifying and classifying nodules and adenocarcinomas. Therefore, pneumonia radiomic signature is not well-established, even for typical pneumonia.

We still do not know how COVID-19 affects the immune system and the lungs, but some cases do not have any CXR alterations, they have only parenchymal abnormalities (75). For further studies, PCR positive COVID-19 individuals without visible CXR modifications should be analyzed using radiomic features to evaluate small regions looking for pulmonary tissue texture variations. In addition, when available, further studies should include clinical information to allow the evaluation of the benefits in diagnosis when using both radiomics and clinical data.

A big challenge in using large CXR image databases is maintaining label information such as projection (i.e., lateral, AP, PA). The further effort in global data curation could confirm projection without the need for visual confirmation.

### Conclusions

This article presents the SHAP approach to explain machine learning classification models based on hand-crafted radiomics texture features to provide grounds for understanding the characteristic radiographic findings on CXR images of patients with COVID-19. The XGB ML model is the best discriminant method between COVID-19 pneumonia and healthy and other lung pathologies using radiomic features extracted from lung CXR images divided into six zones. The explainable model shows the importance of the middle left and superior right lung zone in classifying COVID-19 pneumonia from other lung patterns. The method can potentially be clinically applied as a first-line triage tool for suspected individuals with COVID-19.

The rapid increase of COVID-19 pneumonia cases shows the necessity of urgent solutions to differentiate individuals with and without COVID-19 due to its high spreadability and necessity of prompt management of ill individuals. Furthermore, the lack of knowledge about the disease made it necessary to find explainable radiological features to correlate with the biological mechanisms of COVID-19.

## Data Availability Statement

Publicly available datasets were analyzed in this study. This data can be found here: https://bimcv.cipf.es/bimcv-projects/bimcv-covid19/.

## Author Contributions

LM: study design, data mining and pre-processing, model development, writing, and analysis. CM and CD: study design, literature review, writing, and analysis. RB and AM: study design. All authors contributed to the article and approved the submitted version.

## Funding

This study was financed in part by the Coordenação de Aperfeiçoamento de Pessoal de Nivel Superior – Brasil (CAPES) – Finance Code 001 and PUCRS by financial support with research scholarship.

## Conflict of Interest

The authors declare that the research was conducted in the absence of any commercial or financial relationships that could be construed as a potential conflict of interest.

## Publisher's Note

All claims expressed in this article are solely those of the authors and do not necessarily represent those of their affiliated organizations, or those of the publisher, the editors and the reviewers. Any product that may be evaluated in this article, or claim that may be made by its manufacturer, is not guaranteed or endorsed by the publisher.

## Footnotes

^1^https://github.com/imlab-uiip/lung-segmentation-2D.

^2^https://ing-bank.github.io/probatus/.

## Abbreviations

1st order: First-order features
AP: anteroposterior
AUC: area under the curve
COVID-19: Coronavirus SARS-CoV-2 diseases
CNN: Convolutional Neural Networks
CXR: Chest X-ray
GLCM: Gray-level co-occurrence matrix
GLRLM: Gray-level Run Length Matrix
GLSZM: Gray-level Size Zone Matrix
GLDM: Gray-level Dependence Matrix
PA: posteroanterior
PCR: Protein C-reactive
ROC: receiver operating characteristic
SHAP: Shapley Additive Explanations
SHAP-RFECV: Shapley Additive Explanations with Recursive Feature Elimination with Cross-validation.
